# FGF21 Can Be Mimicked In Vitro and In Vivo by a Novel Anti-FGFR1c/β-Klotho Bispecific Protein

**DOI:** 10.1371/journal.pone.0061432

**Published:** 2013-04-22

**Authors:** Richard Smith, Amy Duguay, Alice Bakker, Peng Li, Jennifer Weiszmann, Melissa R. Thomas, Benjamin M. Alba, Xinle Wu, Jamila Gupte, Li Yang, Jennitte Stevens, Agnes Hamburger, Stephen Smith, Jiyun Chen, Renee Komorowski, Kevin W. Moore, Murielle M. Véniant, Yang Li

**Affiliations:** 1 Therapeutic Discovery, Amgen Inc., South San Francisco, California, United States of America; 2 Metabolic Disorders, Amgen Inc., South San Francisco, California, United States of America; 3 Therapeutic Discovery, Amgen Inc., Thousand Oaks, California, United States of America; 4 Pharmacokinetics and Drug Metabolism, Amgen Inc., South San Francisco, California, United States of America; 5 Metabolic Disorders, Amgen Inc., Thousand Oaks, California, United States of America; University of Texas Health Science Center at San Antonio, United States of America

## Abstract

The endocrine hormone FGF21 has attracted considerable interest as a potential therapeutic for treating diabetes and obesity. As an alternative to the native cytokine, we generated bispecific Avimer polypeptides that bind with high affinity and specificity to one of the receptor and coreceptor pairs used by FGF21, FGFR1c and β-Klotho. These Avimers exhibit FGF21-like activity in in vitro assays with potency greater than FGF21. In a study conducted in obese male cynomolgus monkeys, animals treated with an FGFR1c/β-Klotho bispecific Avimer showed improved metabolic parameters and reduced body weight comparable to the effects seen with FGF21. These results not only demonstrate the essential roles of FGFR1c and β-Klotho in mediating the metabolic effects of FGF21, they also describe a first bispecific activator of this unique receptor complex and provide validation for a novel therapeutic approach to target this potentially important pathway for treating diabetes and obesity.

## Introduction

Fibroblast Growth Factor 21 (FGF21) is one of several members of the fibroblast growth factor (FGF) family that function as endocrine hormones. The three members of this subfamily (the FGF19 subfamily), FGF19, FGF21 and FGF23, have been implicated in regulating cholesterol and bile acid synthesis, glucose and lipid homeostasis, and phosphate and vitamin D metabolism respectively [1]. FGF21 transgenic mice exhibit reduced insulin, serum cholesterol and total triglycerides as well as improved glucose control, insulin sensitivity and resistance to diet-induced weight gain [2], [3]. In contrast, FGF21 knockout mice have impaired glucose tolerance, increased body weight and liver steatosis [4]. In both diabetic rodent and non-human primate models, recombinant FGF21 reduces fasting blood glucose, triglycerides, insulin and glucagon, improves serum lipoprotein profiles and reduces body weight [5]–[9]. Importantly from a safety standpoint administration of FGF21 did not lead to hypoglycemia [5]. These observations have generated considerable interest in FGF21 as a potential therapeutic for treating Type II diabetes [10]–[12].

Although there are many examples of recombinant versions of native proteins being used as therapeutics, their properties may not be ideal from the perspective of drug development. Considerable re-engineering may be necessary to improve pharmacokinetics, solubility, stability and manufacturability. This also appears to be the case for native FGF21 as the wild type protein has poor plasma stability [13]. An alternative approach to re-engineering the native protein is to generate a completely novel protein that has the same pharmacodynamic properties as the original protein whilst having improved drug-like properties.

Unlike canonical fibroblast growth factors, FGF21 does not interact directly with FGF receptors (FGFRs) in association with heparan sulfate. Instead FGF21 requires a single pass transmembrane co-receptor, β-Klotho, to mediate interactions with and activation of FGFRs [14]. β-Klotho is selectively expressed in liver, adipose and pancreas [15]. The strict requirement for β-Klotho to signal limits the site of action for FGF21 to these tissues, despite the widespread expression of FGFRs Out of the 7 major FGFRs, β-Klotho has been shown to interact with FGFR4 and the c isoforms of FGFR1, 2, and 3 [14]. In vitro, FGF21 has been shown to activate three of the β-Klotho partners, FGFR1c, 2c, and 3c complexed with β-Klotho, but not FGFR4. Recent studies using specific activators of β-Klotho/FGFR1c and FGFR1 and β-Klotho knockout mice have demonstrated β-Klotho/FGFR1c as the in vivo receptor complex that mediates the main metabolic effects of FGF21 [16]–[19].

It is generally believed that FGFs act by inducing homodimerization of FGF receptors to activate receptor tyrosine kinase activities [20]. Although our understanding of how FGF21 interacts and activates β-Klotho/FGFR receptor complexes remains incomplete, recent results suggest that β-Klotho may serve primarily as a structural scaffold that docks FGF21 onto the FGF receptor complex. Subsequently, FGF21 may induce receptor dimerization and activation in a manner similar to paracrine FGFs [21], [22]. However, it is not clear what features would be required for a novel molecule to confer FGF21-like activity.

We hypothesized that a bispecific artificial protein that simultaneously binds β-Klotho and its FGFR partner could activate this receptor complex and mimic the activity of FGF21. We generated a series of FGFR1c/β-Klotho bispecific proteins using the Avimer scaffold, which is based on the A-domain, an approximately 4 kDa domain that is involved in mediating protein-protein interactions [23]. Using phage-displayed libraries we generated Avimers that bound specifically to either FGFR1c or β-Klotho. These domains were then combined into a single polypeptide to generate a bispecific molecule that exhibits potent FGF21-like agonist activity in vitro and in vivo.

## Results

### Generation of FGFR1c and β-Klotho-specific Avimer domains

Our goal was to design a bispecific Avimer that binds FGFR1c and β-Klotho, and test whether it elicits an FGF21-like signaling response in target cells. Generation of phage-displayed A-domain libraries has been previously described [23]. These libraries were panned against the recombinant extracellular domains of either human FGFR1c or human β-Klotho. Twenty nine FGFR1c monomers, with binding affinities ranging from 0.15 nM to 25 nM (as measured by ELISA), were isolated. 39 β-Klotho dimers were isolated using the “walking” method of affinity maturation [23]. The β-Klotho dimers had binding affinities ranging from 0.01 nM to 100 nM (as measured by ELISA).

### FGFR1c/β-Klotho bispecific Avimers have agonistic activity in FGF21-responsive in vitro assays

**Figure 1:**
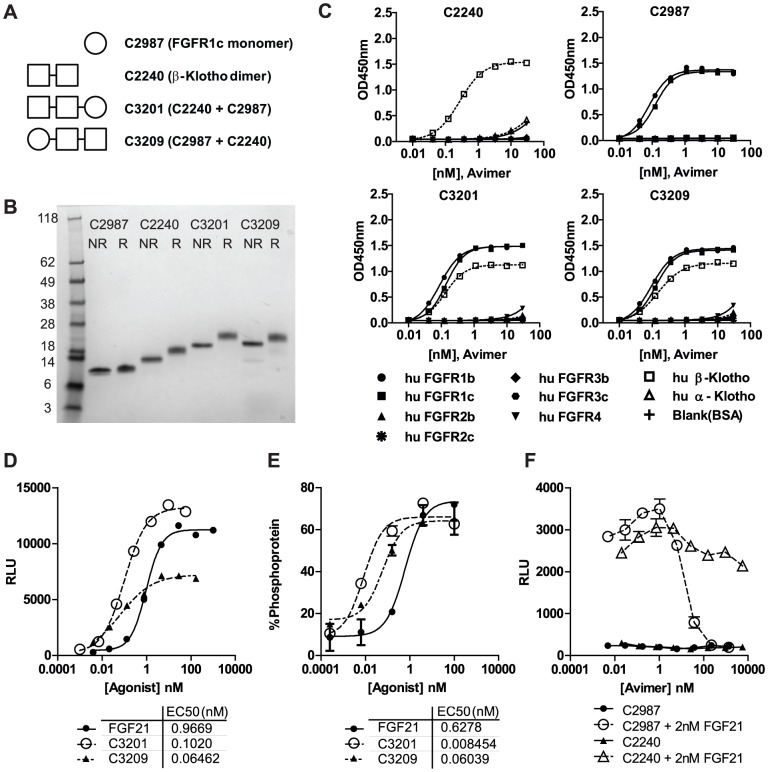
Design and characterization of FGFR1c/β-Klotho bispecific Avimers. (A) Cartoon representation of FGFR1c monomer C2987 and β-Klotho dimer C2240 and orientation of these component Avimers in the bispecific Avimers C3201 and C3209. (B) Purified Avimers C2987, C2240, C3201 and C3209 analyzed by SDS-PAGE. 1 µg of protein was run per lane, with samples either reduced (R) or not reduced (NR). The upward shift on reduction is characteristic of Avimer proteins. (C) ELISA analysis of purified Avimers showing the specificity for both FGFR1c and β-Klotho is retained in the bispecific Avimers C3201 and C3209. Binding is measured by optical density (OD) at 450 nm. (D) Bispecific Avimers C3201 and C3209 induce luciferase activity in an FGF21-responsive engineered reporter cell line following four hour stimulation of serum-starved cells. Luciferase activity is measured in relative luminescence units (RLU). (E) FGF21 and bispecific Avimers C3201 and C3209 induce ERK phosphorylation in cultured primary human adipocytes. Data are expressed as % ERK protein that is phosphorylated, as measured by MSD. (F) Monospecific Avimers C2987 and C2240 were titrated on FGF21-responsive reporter cells +/−2 nM FGF21. The FGFR1c monomer C2987 antagonizes FGF21. Luciferase activity is measured in relative luminescence units (RLU). Data are represented as mean +/− SEM (n = 2).

Various combinations of FGFR1c monomers and β-Klotho dimers were linked together in a single polypeptide chain and tested for agonistic activity in FGF21-responsive reporter cell assays. An FGFR1c-specific monomer (C2987) and a β-Klotho specific dimer (C2240) were fused together in two orientations, with C2240 at the N-terminus (termed C3201) or at the C-terminus (termed C3209) (Figures 1A, 1B and Table 1). C2987 and C2240 were highly specific for their targets as determined by ELISA with binding affinities of 0.2 nM and 0.15 nM respectively. Specificity and affinity were retained in the bispecific Avimers C3201 and C3209 (Figure 1C).

**Table 1:** Selected Avimer amino acid sequences.

**C2240**	**CGADQFRCGNGSCVPRAWRCDGVDDCGDGSDEAPEICETPTCQSNEFRCRSGRCIPQHWLCDGLNDCGDGSDESQQCSAPASEPPGSLSL**
**C2987**	**CGEGLFTCRSTNICISHAWVCDGVDDCEDNSDENNCSAPASEPPGSL**
**C3201**	**CGADQFRCGNGSCVPRAWRCDGVDDCGDGSDEAPEICETPTCQSNEFRCRSGRCIPQHWLCDGLNDCGDGSDESQQCSAPASEPPGSLCGEGLFTCRSTNICISHAWVCDGVDDCEDNSDENNCSAPASEPPGSL**
**C3209**	**CGEGLFTCRSTNICISHAWVCDGVDDCEDNSDENNCSAPASEPPGSLCGADQFRCGNGSCVPRAWRCDGVDDCGDGSDEAPEICETPTCQSNEFRCRSGRCIPQHWLCDGLNDCGDGSDESQQCSAPASEPPGSL**

The orientation of binding domains is important. FGFR1c/β-Klotho bispecific Avimers were tested for agonistic activity using an FGF21-responsive reporter cell assay. C3201 is a full agonist with ten-fold greater potency than FGF21 in the reporter cell assay. In contrast C3209, whilst similarly potent to C3201, is a partial agonist in the same assay (Figure 1D). Nonetheless, both C3201 and C3209 are full agonists on primary human adipocytes, the putative FGF21 target cell population, as measured by induction of ERK phosphorylation. In this assay C3201 and C3209 are 74-fold and 10-fold more potent than FGF21 respectively (Figure 1E). The monospecific constituent binding domains C2987 and C2240 do not have any agonistic activity, though the FGFR1c binding monomer C2987 does antagonize FGF21. C2240 does not antagonize FGF21 (Figure 1F).

### C3201 binding epitopes

**Figure 2:**
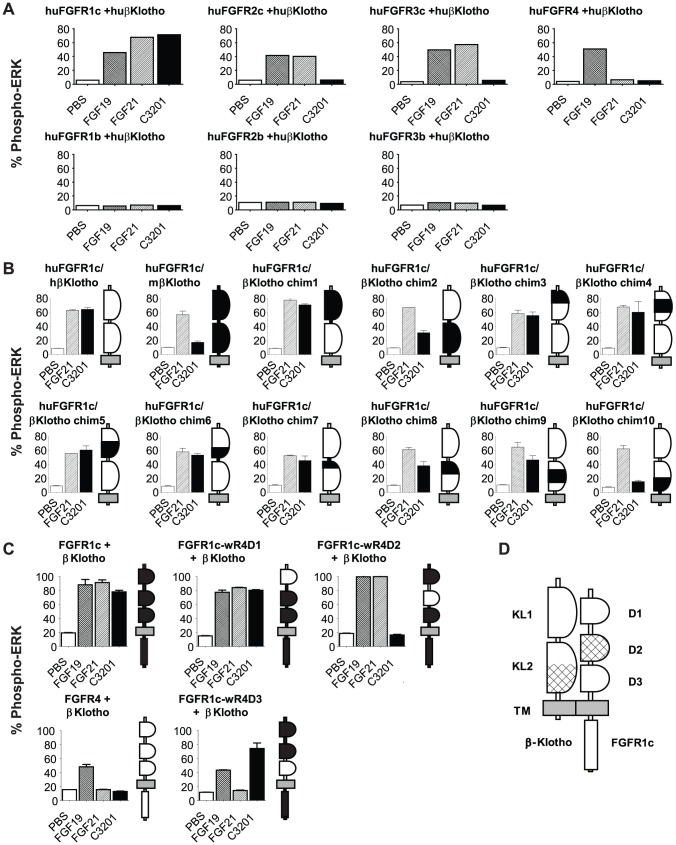
Characterization of binding sites for C3201 on FGFR1c and β-Klotho. (A) C3201 requires FGFR1c and β-Klotho to induce signaling as measured by ERK phosphorylation in L6 cells. L6 cells were selected for this characterization as they do not express any endogenous FGF receptors. Data are expressed as % ERK protein that is phosphorylated, as measured by MSD. (B) Characterization of C3201 binding site on β-Klotho. C3201 does not bind to murine β-Klotho. A series of human-mouse β-Klotho chimeras were co-transfected into L6 cells with human FGFR1c, and signaling was measured by ERK phosphorylation. The β-Klotho chimeras are represented by cartoons next to the ERK phosphorylation histograms, with murine sequence indicated as black and human sequence represented as white. The plasma membrane is indicated in grey. Data are represented as mean +/− SEM (n = 2). (C) Characterization of C3201 binding site in FGFR1c. C3201 does not signal through FGFR4. A series of FGFR1c/FGFR4 domain swap mutants were constructed and transiently transfected into L6 cells with human β-Klotho, and signaling was measured by ERK phosphorylation. The FGFR1c/FGFR4 domain swap mutants are represented by cartoons next to the ERK phosphorylation histograms, with FGFR1c sequences indicated as white and FGFR4 sequences represented as black. The plasma membrane is indicated in grey. Data are represented as mean +/− SEM (n = 2). (D) Predicted location of C3201 binding sites in the FGFR1c/β-Klotho complex. The predicted binding sites are indicated by cross-hatched areas on cartoon in FGFR1c domain D2 and the C-terminal region of β-Klotho domain KL2.

Based on its activity in cell-based assays bispecific Avimer C3201 was selected for further characterization. C3201 required coexpression of FGFR1c and β-Klotho to induce signaling in transiently transfected L6 cells, as measured by induction of ERK phosphorylation (Figure 2A). In contrast, FGF21 can signal through FGFR1c, FGFR2c and FGFR3c when these receptors are co-expressed with β-Klotho on L6 cells (Figure 2A). Although C3201 can bind to FGFR1b (Figure 1C), it cannot induce signaling through FGFR1b. This may reflect an inability of the ‘b’ isoforms of the FGF receptors to form a heterodimeric complex with β-Klotho [14], [24].

We attempted to identify the regions on FGFR1c and β-Klotho that bind to C3201 using a series of domain swap mutants of each target protein. ELISA data (not shown) indicated that C3201 and its constituent β-Klotho binding dimer C2240 bind to human but not mouse β-Klotho. Human FGF21 can bind to both human and mouse β-Klotho. A series of human-mouse β-Klotho chimeric proteins were constructed and transiently transfected with human FGFR1c into L6 cells (Table 2). As predicted by the ELISA data, C3201 did not induce signaling in cells expressing murine β-Klotho, while human FGF21 was active in the presence of either human or mouse β-Klotho. C3201 induced signaling when all or part of human β-Klotho domain 1 was replaced with murine sequence. In contrast, C3201-induced signaling was reduced when human β-Klotho domain 2 was replaced with murine sequence, suggesting that C3201 binds principally to β-Klotho domain 2 (Figure 2B). β-Klotho chimeras 7 through 10 allowed a more precise localization of the C3201 binding site to the C-terminal portion of β-Klotho domain 2, as replacement of this region with murine sequence in chimera 10 reduced C3201-induced signaling (Figure 2B). Reporter cell assay data indicate that the β-Klotho binding dimer C2240 does not antagonize FGF21 signaling, implying that the binding sites for C3201 and FGF21 on β-Klotho are distinct (Figure 1F).

**Table 2:** Human/mouse β-Klotho chimeras used to determine Avimer binding site.

Chimera	Primary Structure
1	*1-F519 (Mouse)*::**P522-S1044** (Human)
2	**1-F521 (Human)**::*P520-S1043 (Mouse)*
3	**1-F82 (Human)**::*P83-G301 (Mouse)*::**S302-S1044 (Human)**
4	**1-L193 (Human)**::*Y194-N415 (Mouse)*::**G418-S1044 (Human)**
5	**1-G301 (Human)**::*S302-F506 (Mouse)*::**S509-S1044 (Human)**
6	**1-N417 (Human)**::*G416-F519 (Mouse)*::**P522-S1044 (Human)**
7	**1-F506 (Human)**::*G507-G632 (Mouse)*::**L635-S1044 (Human)**
8	**1-F521 (Human)**::*P520-A735 (Mouse)*::**V738-S1044 (Human)**
9	**1-L633 (Human)**::*G632-Q849 (Mouse)*::**D852-S1044 (Human)**
10	**1-G736 (Human)**::*A735-S963 (Mouse)*::**G967-S1044 (Human)**

Similarly C3201 and its constituent FGFR1c binding monomer C2987 were shown by ELISA to bind FGFR1c but not FGFR4 (Figure 1C). A series of FGFR1c/FGFR4 chimeras were generated (Table 3) and transiently transfected with human β-Klotho into L6 cells. C3201 and FGF21 were able to signal through FGFR1c/β-Klotho but not FGFR4/β-Klotho, as measured by ERK phosphorylation. The integrity of the FGFR4 construct was confirmed by FGF19, which was able to signal through both FGFR1c and FGFR4 as previously reported [25]–[27]. FGF21 signaling was eliminated when the linker between D2 and D3 plus D3 of FGFR1c was replaced with the D2–D3 linker and D3 of FGFR4 (Figure 2C). This supports previously published work suggesting that members of the FGF family bind to their cognate receptors at the junction of D2 and D3 [22], [28]. In contrast C3201 appears to interact entirely within D2 (Figure 2D). Although the domain swap mutants indicate that C3201 and FGF21 do not bind at precisely the same site, the reporter cell assay indicates that the FGFR1c binding domain C2987 does antagonize FGF21 (Figure 1F). These data suggest that although FGF21 and C3201 do not bind exactly the same epitope in FGFR1c, their binding sites are close enough to result in C3201 being able to sterically hinder FGF21 binding to FGFR1c.

**Table 3:** FGFR1c/FGFR4 chimeras used to determine Avimer binding site.

Chimera	Primary Structure
FGFR1c-wR4D1	*1-Q145(R4)* :: **A146-R816(R1c)**
FGFR1c-wR4D2	**1-T141(R1c)** :: *R142-S248(R4)* :: **P249-R819(R1c)**
FGFR1c-wR4D3	**1-N264(R1c)** :: *T265-L357(R4)* :: **E358-R821(R1c)**

### Fusing C3201 to human serum albumin extends serum half-life in cynomolgus monkeys

**Figure 3:**
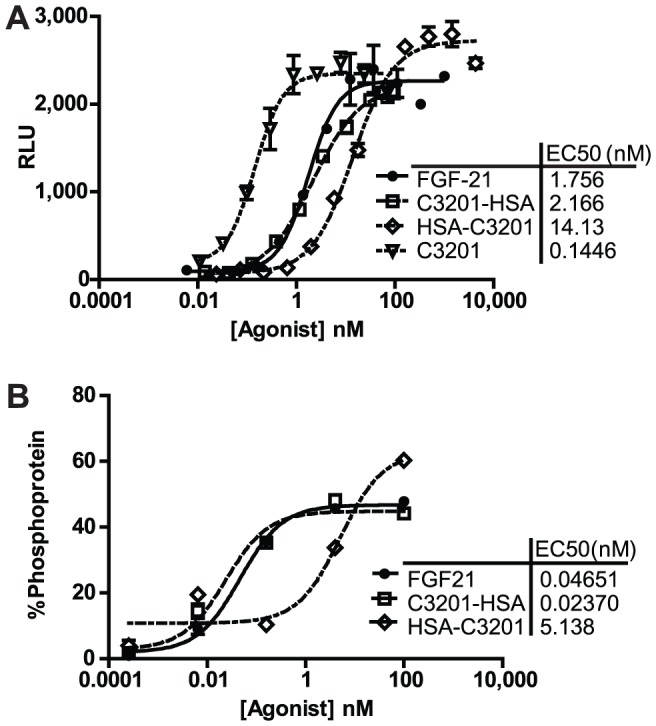
Characterization of C3201-HSA fusions. (A) Fusions of C3201 and HSA induce luciferase activity on an FGF21-responsive engineered reporter cell line following four hour stimulation of serum-starved cells. Potency is influenced by the orientation of the fusion. Luciferase activity is measured in relative luminescence units (RLU). Data are represented as mean +/− SEM (n = 2). (B) Fusions of C3201 and HSA induce ERK phosphorylation in cultured primary human adipocytes. Potency is influenced by the orientation of the fusion. Data are expressed as % ERK protein that is phosphorylated, as measured by MSD.

C3201 is approximately 18 kDa in size, rendering it small enough to be rapidly excreted from the kidneys. Therefore we chose to improve its pharmacokinetics by generating a C3201-human serum albumin (HSA) fusion protein. In addition to preventing excretion from the kidneys, the addition of HSA allows serum half-life to be extended by recycling through the endothelial FcRn pathway [29]–[32]. C3201 was linked to either the N- or C-terminus of HSA with the resulting fusion proteins being expressed by stably transfected Chinese Hamster Ovary (CHO) cells. We found that fusing C3201 to HSA reduced the potency of C3201 (Figure 3A). However this effect was minimal when C3201 was fused to the N-terminus of HSA (C3201-HSA). When C3201 was fused to the C-terminus of HSA (HSA-C3201) there was a greater loss in potency (Figure 3A). In addition to being active on FGF21-responsive reporter cells, C3201-HSA induced ERK phosphorylation in cultured primary human adipocytes with potency and efficacy comparable to native FGF21 (Figure 3B).

The pharmacokinetics of C3201-HSA was determined in lean male cynomolgus monkeys. C3201-HSA was administered as a single intravenous dose at 1 mg/kg, and was found to have a terminal half-life of 50 hours (Figure S1A). A subsequent single subcutaneous dose PK study indicated C3201-HSA has a bioavailability of 53% by the subcutaneous route (Figure S1B), the favored mode of administration for functional testing.

### C3201-HSA induces FGF21-like effects in obese cynomolgus monkeys

**Figure 4:**
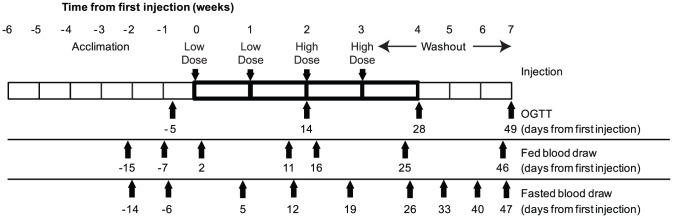
Design of C3201-HSA functional study. The study consisted of a four week acclimation period, four once weekly doses of C3201-HSA and a three week wash out period. Dates for collecting blood draws are indicated. These samples were tested for blood chemistry, C3201-HSA concentration and presence of anti-drug antibody. Body weight was determined once per week on the day that the animals were dosed. Forty animals were randomly assigned to four groups of ten. Group one was treated with Avimer vehicle (20 mM Tris-HCl, pH 7.4, 150 mM NaCl, 1 mM CaCl2). Group two was treated with a low dose of C3201-HSA (0.5 mg/kg/week, weeks 1 and 2, 1 mg/kg/week, weeks 3 and 4). Group three was treated with a high dose of C3201-HSA (2.5 mg/kg/week, weeks 1 and 2, 5 mg/kg/week, weeks 3 and 4). Group four was treated with of a positive control long-acting FGF21 variant Fc-FGF21 with a 3 mg/kg dose once per week.

C3201 did not bind to murine β-Klotho and was not active in murine adipocytes but showed in vitro activity in luciferase reporter cells transfected with cynomolgus monkey β-Klotho and human FGFR1c (which has the same amino acid sequence as cynomolgus monkey FGFR1c). Therefore we tested its in vivo function in obese cynomolgus monkeys. These monkeys had body mass indices (BMI) ranging from 32 to 74 kg/m^2^ but presented normal plasma glucose, insulin and triglycerides levels at baseline. The pharmacokinetic profile assessed in lean cynomolgus monkeys allowed us to choose two dose escalation regimens; 0.5 mg/kg/week for two weeks followed by 1 mg/kg/week for another two weeks (based on adipocyte assay EC90) and 2.5 mg/kg/week followed by 5 mg/kg/week for another two weeks (based on reporter assay EC90) (Figure 4). Plasma concentration of C3201-HSA was maintained above the EC_90_ of the reporter cell assay (10 nM) for the high dose group and above the EC_90_ of the primary adipocyte assay (1 nM) for the low dose group. In addition, a long-lived variant of FGF21 fused to Fc (Fc-FGF21) dosed at 3 mg/kg/week was used as a positive control [13]. All dosing regimens were preceded by a six week acclimation period. Baseline values for body weight and blood chemistry were determined towards the end of the acclimation period (Table 4).

**Table 4:** PD parameters at baseline and 2 weeks after first administration of low and high doses of C3201-HSA and vehicle.

	Vehicle			C3201-HSA Low			C3201-HSA High		
	Baseline	2 weeks	% Change	Baseline	2 weeks	% Change	Baseline	2 weeks	% Change
Body Weight (kg)	8.65±0.39	8.66±0.42	**0.1%**	9.17±0.5	8.61±0.48	**−6.1%**	8.8±0.79	**8.08±0.75** #	**−8.2%**
BMI (kg/m^2^)	51.3±1.9	49.78±1.91	**−3.0%**	48.76±3.13	45.71±2.86	**−6.3%**	46.84±4.3	43.07±4.19	**−8.0%**
Insulin (Fasting) (ng/ml)	2±0.55	3.29±0.9	**64.5%**	2.4±0.51	**2.2±0.48** #	**−8.3%**	2.2±0.46	**2.03±0.46** #	**−7.7%**
Insulin (Post-feeding) (ng/ml)	3.86±0.78	4.97±1.85	**28.8%**	4.44±1.05	3.91±1.97	**−11.9%**	3.29±0.72	**2.06±0.29** #	**−37.4%**
Triglyceride (Fasting) (mg/dl)	109.9±25.1	100±26.4	**−9.0%**	113.9±15.2	**71.86±14** #	**−36.9%**	113.9±24.8	**58.14±14.3** #	**−49.0%**
Triglyceride (Post-feeding) (mg/dl)	93±19	110±26	**18.3%**	114.8±17.6	**68±17.98** #	**−40.8%**	119.5±26	**59±12** #	**−50.6%**
HDL (mg/dl)	59±2.48	56.12±2.87	**−4.8%**	59.52±3.87	59.19±3.21	**−0.6%**	58.1±2.05	60.77±1.86	**4.6%**
Abdominal Circumference (m)	0.46±0.01	0.42±0.01	**−8.7%**	0.46±0.02	0.41±0.03	**−10.9%**	0.45±0.04	0.4±0.04	**−11.1%**
Skin Fold Thickness (cm)	8.24±1	8.68±0.94	**6.9%**	8.2±1.92	7.53±1.67	**−8.2%**	9.53±3.25	9.23±2.98	**−3.1%**

The values (± SEM) shown are the average measurements made on all ten animals in each group. The percentage changes after two weeks relative to baseline are indicated for each group.

# indicates value significantly varies from equivalent 2 week value in Vehicle group (p<0.05).

**Figure 5:**
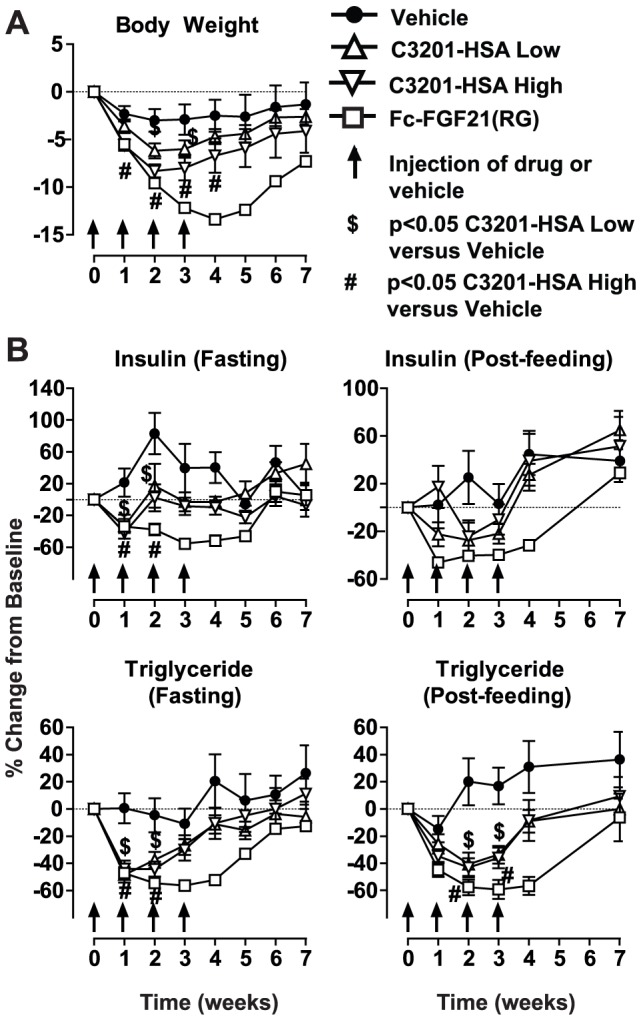
Pharmacodynamic properties of C3201-HSA in obese cynomolgus monkeys. (A) Changes in body weight following administration of vehicle, low or high dose of C3201-HSA or Fc-FGF21. (B) Changes in plasma insulin and triglycerides measured after feeding and overnight fast following administration of vehicle, low or high dose of C3201-HSA or Fc-FGF21. Data are represented as mean +/− SEM (n = 10).

Body weight was monitored weekly throughout the study. Over the course of the four week treatment, the body weight of animals treated with vehicle remained constant while body weight of animals treated with C3201-HSA (low and high doses) and Fc-FGF21 progressively decreased. In these groups, body weight nearly returned to baseline by the end of the wash out period (Figure 5A).

Blood was collected from animals after overnight fast or two hours after the morning meal. As expected, C3201-HSA did not induce hypoglycemia. Also, as these monkeys were normoglycemic, fasting or fed plasma glucose levels after treatment with C3201-HSA were not significantly decreased (data not shown). However, C3201-HSA treatment resulted in a statistically significant decrease in fasting plasma insulin levels and a trend towards decreased fed plasma insulin levels (Figure 5B). Moreover, after both fasting and feeding, triglyceride levels were significantly reduced in animals treated with C3201-HSA (Figure 5B) for the first two weeks. These changes were also observed with Fc-FGF21.

**Figure 6:**
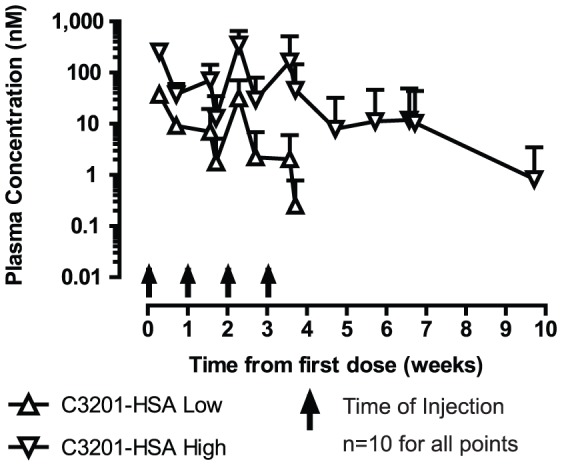
Analysis of C3201-HSA serum levels in C3201-HSA in vivo functional study. Mean serum concentration of C3201-HSA in low and high dose groups determined by specific sandwich ELISA. N = 10 for both groups. Data are represented as mean +/− SEM (n = 10).

The majority of parameters that were measured showed greatest changes from baseline 2 weeks after the first dose (Table 4). All the parameters that showed significant changes when compared to the vehicle group in animals treated with C3201-HSA started to return back to baseline while the animals were still receiving treatment. Pharmacokinetic analysis of samples from the treated animals detected C3201-HSA in serum throughout the treatment period, with spikes in serum concentration corresponding to each dose (Figure 6). However, anti-drug antibodies (ADA) were detected in later samples (taken after 12 days). The ADA response was directed predominantly to the Avimer portion of C3201-HSA, with relatively lower titers against HSA. ADA did not significantly bind to an irrelevant control Avimer (Figure S2A). The ADA response neutralized C3201 activity in the reporter cell assay. However, serum from animals treated with vehicle did not affect C3201 activity (Figure S2B).

## Discussion

There is increasing evidence to suggest the utility of FGF21 as a therapeutic for treating diabetes and obesity. There are challenges involved in using a native protein as a therapeutic, particularly one with a short half-life such as FGF21. Half-life can be extended by conjugation to polymers such as polyethylene glycol (PEG) [33]–[35], though this adds to the complexity and cost of the manufacturing process and there is evidence that some PEGylated proteins can induce formation of vacuoles in the kidney [36]. In addition, native proteins may require re-engineering in order to give them optimal drug-like properties [37]. These challenges can be circumvented by using a completely novel therapeutic protein that does not have any sequence homology with the native protein yet induces a comparable biological response. For FGF21 this required generating a protein that could, like FGF21, bind the co-receptor β-Klotho and signals through FGFR1c. This concept was first validated using a novel agonistic antibody against this receptor complex [17]. Here, we took a different approach by constructing a protein using the Avimer scaffold that can bind simultaneously to both FGFR1c and β-Klotho to explore potential novel therapeutic and to further demonstrate the central role of this receptor complex in FGF21 function.

The induction of signaling by FGF21 is a complex process resulting from the formation of a ternary complex between FGFR1c, β-Klotho and FGF21. FGFR1c can remain inactive in close proximity to β-Klotho in the absence of FGF21 [38], [39]. When FGF21 and β-Klotho are bound together they are able to dimerize FGFR1c in a way that allows the activation of signaling [21]. Several different approaches could be pursued to induce receptor dimerization, such as using a bivalent molecule that cross-links the receptors; for example, having both arms of the bivalent molecule bind to β-Klotho or FGFR1c alone. However, such an approach could result in either activation of multiple FGFRs that are capable of partnering with β-Klotho or global activation of FGFR1 independent of β-Klotho, potentially resulting in undesirable side-effects from either lack of FGFR1c/β-Klotho specificity or mitogenicity. For example, while an agonistic anti-FGFR1 monoclonal antibody was shown to induce FGF21-like activity in rodents, it also induced a reduction in serum inorganic phosphate, an FGF23 response that resulted from the lack of β-Klotho dependence [40]. In addition, the lack of β-Klotho dependence also raises important questions around potential antibody-induced mitogenicity due to global activation of FGFR1 alone. Therefore, to obtain a molecule that is dependent on both β-Klotho and FGFR1c for signaling, we pursued a bispecific molecule with one arm binding to β-Klotho and the other binding to FGFR1c. We generated binders to both FGFR1c and β-Klotho using phage-displayed A-domain libraries. The modular nature of the Avimer technology allowed us to link binders to FGFR1c and β-Klotho into single bispecific polypeptides and rapidly screen them for FGF21-like activity in cell-based assays. Our screening identified that fusing a monomeric Avimer, C2987, that specifically bound to FGFR1c, to a β-Klotho specific dimeric Avimer, C2240, resulted in a bispecific molecule, termed C3201 (Figure 1A), that was highly potent in FGF21 sensitive cell-based assays.

The FGFR1c-binding monomeric Avimer C2987 binds to FGFR1cD2. Although it has been shown that FGF21 binds to FGFR1cD3 [22], the Avimer C2987 binding site is in close enough proximity to the binding site for FGF21 that the Avimer can antagonize FGF21 (Figure 1F). The β-Klotho binding dimeric Avimer C2240 binds the C-terminal portion of β-Klotho domain KL2 (Figure 2B). This does not coincide with the binding site for FGF21, as C2240 does not antagonize FGF21 in the reporter cell assay (Figure 1F). Although C3201 does not bind to FGFR1c or β-Klotho in exactly the same locations as FGF21 it was still able to elicit signaling similar to FGF21. While these data suggest that there are multiple ways by which a ligand can bring FGFR1c and β-Klotho together to form an active signaling complex it is also important to note that the orientation of the FGFR1c and β-Klotho binding domains in the Avimer are critical for full agonist activity (Figure 1D). A significant difference between C3201 and FGF21 is that C3201 can bind FGFR1c with sub-nanomolar affinity in the absence of β-Klotho unlike FGF21 which is unable to bind FGFR1c alone [14], [24]. Yet, C3201 is unable to elicit signaling through FGFR1c alone. Like FGF21, C3201 requires the presence of β-Klotho in order to elicit signaling, supporting the hypothesis that bringing β-Klotho into an appropriate interaction with FGFR1c is necessary for FGF21 signaling.

The Avimer described here, C3201, required modification to provide suitable serum half-life for in vivo characterization. The modular nature of the Avimer domain allows the incorporation of a domain that binds to a long-lived serum protein such as serum albumin or IgG [23]. This approach has also been described for a number of other non-IgG protein scaffolds (reviewed in [34]). For the purposes of the current study we constructed a genetic fusion of C3201 with HSA. The resulting protein was stable, retained FGF21-like activity and could be expressed at >1 g/L from a stable CHO cell line. This level of expression is comparable to that seen for commercialized therapeutic proteins. The relative ease with which such fusions can be made has led to considerable interest in HSA fusions as therapeutic molecules. In our hands, C3201-HSA had a half-life sufficient for once weekly dosing. Under these conditions, male obese cynomolgus monkeys treated with C3201-HSA showed improved metabolic parameters. Body weight was reduced. Insulin and triglyceride levels were also reduced by C3201-HSA in fed and fasting states. These metabolic changes are similar to the effects seen in rodents and non-human primates treated with FGF21 [5]–[8], [41]–[43] and were also observed in the current study with our positive control molecule, the long-acting FGF21 variant Fc-FGF21 [13]. These results further supports the concept that β-Klotho/FGFR1c receptor complex mediates the main metabolic functions of FGF21.

It is not unexpected to see induction of anti-drug antibodies (ADA) when administering multiple doses of a protein in a preclinical model. Animals treated with C3201-HSA had a rapid, high-titer, neutralizing ADA response, predominantly to the Avimer portion of C3201-HSA. The ADA did not recognize an unrelated Avimer, suggesting that this is not a result of a structural motif common to all Avimers. This led to a loss of pharmacodynamic response in the C3201-HSA treatment groups during the dosing phase of the study. We did not observe this loss in the Fc-FGF21 positive control group. Although the induction of ADA raises challenges for performing preclinical studies, these hurdles are not insurmountable and are not necessarily predictive of what may happen in humans [44].

This study demonstrates that a bispecific molecule that binds FGFR1c and β-Klotho can mimic the activity of FGF21 in vitro and in vivo. We showed that our novel bispecific molecule, C3201, was highly specific for FGFR1c in vitro, unlike FGF21 which was able to induce signaling through FGFR1c, 2c and 3c in the presence of β-Klotho in vitro (Figure 2A). Although C3201 only signals through FGFR1c in the presence of β-Klotho, it was still capable of inducing a similar physiological response to FGF21 in cynomolgus monkeys, thus providing more evidence for the role of FGFR1c as receptor for FGF21 and the vital role β-Klotho plays in FGF21 function. This study also has significant implications for the development of biologic therapeutics, allowing the generation of biologically active molecules with no sequence or structural relationship to native proteins and a potentially greater degree of utility and safety.

## Methods

### Ethics Statement

All experimental animal procedures were approved by the Institutional Animal Care and Use Committee of Yunnan Laboratory Primate Inc. China (permit number 2010012106). Animals were cared for in accordance to the *Guide for the Care and Use of Laboratory Animals, 8^th^ Edition* [45]. Animal housing conditions were approved by the Science Technology Agency of Yunnan, China. Animals were single-housed indoors, fed a certified primate diet (SCXK (Yunnan) 2010-0004, Weimin animal husbandry Xishuangbanna Co., Ltd.) and had ad libitum access to water. Food was enriched by providing peanuts, fresh apples or biscuit. Animals were maintained on a 12∶12 hour light: dark cycle. Room temperature was maintained between 18 to 26°C and the humidity between 40–80%. Animal rooms were enriched by providing artificial plants that were exchanged every week. Television was provided twice a week in each room and toys were supplied. Monkeys were acclimated for 6 weeks prior to the initiation of compound administration. During the acclimation period, the monkeys were familiarized with study-related procedures, including chair-restraint, subcutaneous injection, gavage and blood draws.

### Phage Display

Avimer libraries were constructed in the fUSE5 M13 phage vector (provided by George Smith, University of Missouri) and panned against immobilized protein targets as previously described [23]. To derive high affinity FGFR1c binding clones, we isolated a pool of 39 monomers by panning the A-domain library for three rounds against purified recombinant human FGFR1c extracellular domain. Three monomers from this pool were selected for affinity maturation via random mutagenesis. Overlapping oligonucleotides encoding each of the three FGFR1c monomers were synthesized. Scaffold residues [23] were fixed and variable residues were encoded by base doping (91% parental base, 3% for each of the other three bases. The overlapping oligonucleotides were annealed via a fixed nine base pair overlap at 30°C then extended to create double-strand DNA by PCR with LA Taq polymerase (Takara). The assembled double-stranded library oligonucleotides were then cloned into the fUSE5HA phage vector. The library was transformed into EC100 *E.coli* and the resulting phage was panned against human FGFR1c extracellular domain. 29 monomers with improved affinity were isolated. To derive high affinity β-Klotho binding clones we isolated a pool of 57 monomers by panning the A-domain library for three rounds against purified recombinant human β-Klotho and screening for specific β-Klotho binding. A new phage library was generated by fusing a library of random A-domains to either the N- or C-terminus of 8 β-Klotho binding monomers. This library was then panned and screened against β-Klotho as above. 39 dimers with improved affinity were identified. Affinities and specificity were measured by ELISA. Target proteins were coated on a MaxiSorp 96-well plate (Becton Dickinson) at 20 nM overnight at 4°C. The Avimers to be tested were titrated in 20 mM Tris-HCl (pH 7.4), 150 mM NaCl, 1 mM CaCl_2_ and applied to the coated plate. Bound Avimer was detected via an N-terminal influenza hemagglutinin tag using an anti-tag antibody conjugated to horseradish peroxidase (Roche). The plates were developed using TMB chromogenic substrate (Pierce).

### Generation of Bispecific Avimers

FGFR1c and β-Klotho specific Avimers were linked together as multimers in single polypeptide chains and expressed and purified as tagged proteins using methods previously described [23]. For the purposes of initial characterization and purification the bispecific Avimers have an N-terminal influenza hemagglutinin tag and a C-terminal 8X Histidine tag.

### Reporter Cell Assay

Luciferase reporter assays were performed in Chinese Hamster Ovary (CHO) cells stably transfected with human FGFR1c, β-Klotho and reporter constructs containing 5xUAS luciferase and GAL4 DNA binding domain fused to Elk1. In this system luciferase expression is regulated by signaling through endogenous phosphorylated ERK. The CHO reporter cells were plated at 3×10^4^ cells/well on white poly-D-lysine coated plates in Dulbecco's Modified Eagles Medium (DMEM) supplemented with 10% Fetal Bovine Serum (FBS). The next day the medium was replaced with starvation medium (Ham's F12+1% bovine serum albumin) and the cells were incubated for a further 24 hours. Following starvation FGF21 or Avimer proteins were added to the cells. The plates were incubated for 4 hours after which the cells were lysed to measure luciferase activity with the Steady-Glo luciferase assay system (Promega). Luciferase activity was measured in relative luminescence units (RLU).

### Primary Adipocyte Assay

Cultured primary human adipocytes were derived from cryopreserved subcutaneous human preadipocytes (Zen-Bio) according the supplier's instructions. On Day 17 of differentiation, all medium was removed from wells and each well was washed twice with 2 ml of warm PBS. The adipocytes were serum starved for 3 hours in adipocyte serum starvation medium comprised of 1∶1 low glucose DMEM (Invitrogen) and Ham's F12 (Invitrogen) with 0.2% Fatty Acid-Free BSA (Sigma). After 3 hours incubation, 1.4 ml medium was removed and the cells treated with FGF21 or Avimer protein for 10 minutes at 37°C. After 10 minutes treatment, medium was aspirated from wells and the cells washed twice with 2 ml cold PBS. Cells in each well were lysed in 60 µL complete lysis buffer and total and phosphorylated ERK were measured using an MSD whole cell lysate Phospho-ERK1/2 kit (Meso Scale Discovery) according to the manufacturer's instructions.

### L6 Assay

L6 cells were maintained in DMEM supplemented with 10% FBS. Cells were transfected with expression vectors using the Lipofectamine 2000 transfection reagent (Invitrogen) according to the manufacturer's protocol. L6 cells plated in 24-well plates (10^6^ cells/well) were transfected with expression vectors for various FGF receptors and α-Klotho/β-Klotho and serum starved in 0.2% BSA overnight before treatments. Medium was aspirated 10 minutes after treatment and plates were snap-frozen in liquid nitrogen. Cells in each well were lysed in 60 µL complete lysis buffer and total and phosphorylated ERK were measured using an MSD whole cell lysate Phospho-ERK1/2 kit (Meso Scale Discovery) according to the manufacturer's instructions.

### Generation of C3201-HSA fusion

C3201-HSA was expressed from a stably transfected Chinese Hamster Ovary (CHO) cell line. Conditioned medium was concentrated by UFDF and the protein captured with Mimetic Blue chromatography. Following a Q HP chromatography polishing step, C3201-HSA was formulated to a concentration of 82 mg/ml (1 mM) in Tris-buffered saline (20 mM Tris-HCl pH 7.4, 150 mM NaCl) supplemented with 1 mM CaCl_2_.

### Measurement of serum C3201-HSA and pharmacokinetic analysis

A total of four lean female cynomolgus monkeys were assigned to two treatment groups (n = 2/group) and received a single IV injection of C3201-HSA at 1 mg/kg or SC injection of C3201-HSA at 2 mg/kg, respectively (MPI Research). Intensive blood samples were collected up to two weeks. Serum levels of C3201-HSA were measured using a sandwich ELISA format with a lower limit of quantification of 3 ng/mL. A monoclonal Fab fragment specific to the β-Klotho binding Avimer C2240 was generated by phage display (Antibodies by Design Serotec). This Fab was used to capture C3201-HSA from serum samples. The captured C3201-HSA was detected with an unpurified C2240-specific rabbit polyclonal serum (Covance) and an anti-rabbit IgG-horseradish peroxidase conjugate. Serum C3201-HSA concentration-time data were analyzed by noncompartmental methods using WinNonlin Enterprise v 5.1.1 for PK parameters.

### In vivo activity

The study was conducted in obese male cynomolgus monkeys (Macaca fascicularis) at Yunnan Laboratory Primate Inc. The monkeys were 8–15 years old. Their body weights ranged from 5.6 to 13.7 kg and BMI ranged from 32–74 kg/m^2^. Monkeys were acclimated for 6 weeks prior to the initiation of compound administration. During the acclimation period, the monkeys were familiarized with study-related procedures, including chair-restraint, subcutaneous injection (PBS, 0.1 ml/kg), gavage (water, 10 ml/kg), and blood drawn for non-OGTT and OGTT samples. After 4 weeks of training, baseline OGTT and plasma metabolic parameters were measured. 40 monkeys were selected and randomized into one Avimer vehicle (n = 10), two treatment groups (C3201-HSA low dose and C3201-HSA high dose, both n = 10) and one positive control group (n = 10) to achieve similar baseline levels of body weight, glucose OGTT profiles, and plasma glucose and triglyceride levels. To provide a positive control an Fc fusion of FGF21 (Fc-FGF21) with extended serum half life was used [13]. Animals were fed twice a day, with each animal receiving 100 g of formulated food. The remaining food was removed and weighed after each meal to calculate food intake. The feeding times were from 8:00 AM to 8:30 AM (±30 minutes) and then from 4:30 PM to 5:00 PM (±30 minutes). Fruit (apple, 150 g) was supplied to each animal at 1:00 PM to 2:00 PM every day.

The study was conducted in a blinded fashion. Compound was administered weekly by subcutaneous injection at 0.5 mg/kg for the first 2 weeks for C3201-HSA low dose and 2.5 mg/kg for C3201-HSA high dose and at 1 mg/kg for the last 2 weeks for C3201-HSA low dose and 5 mg/kg for C3201-HSA high dose. The positive control Fc-FGF21 was administered weekly at 3 mg/kg for four weeks. After 4 injections of C3201-HSA or Fc-FGF21, animals were monitored during an additional 6 weeks for compound washout and recovery from treatments. Food intake, body weight, clinical chemistry and OGTT were monitored throughout the study. Food intake was measured every meal. Body weight and Body Mass Index (BMI) were monitored weekly throughout the study, both pre- and post-administration of test compound when the body weight was taken. BMI is defined as the individual's body weight divided by the square of his or her crown-rump length. Blood samples were collected on different days in fasted or fed state to measure glucose, insulin and triglyceride levels. In the fasted conditions, blood draws were conducted weekly 5 days after each injection. In the fed conditions (two hours after morning meal), blood draws were conducted on days 2, 11, 16, 25 and 46 post first injection. OGTTs were conducted every two weeks after the initiation of the study. The first day of treatment is designated as day 0 (Figure 4).

Statistical analyses were performed for each metabolic end point and for each post-dose study day independently. SAS version 9.1 (SAS Institute, Inc. Cary, NC) was used. The data were log-transformed prior to statistical analyses. For body weight, fed and fasted blood chemistry parameters, statistical comparison of the means among the groups was made using one-way analysis of covariance model with baseline as a covariate. The 2-sided p-values of comparisons of treatments to vehicle were reported together with estimates of treatment effects and the corresponding 95% confidence intervals. The Sidak's multiple comparison correction was used to yield adjusted p-values in order to maintain the family-wise type I error rate across at 5%.

Serum levels of C3201-HSA were measured as described above. ADA was detected using an ELISA format, with C3201, C3201-HSA, HSA or an unrelated Avimer (C2648) directly coated to a 96 well plate (Maxisorp, Becton Dickinson). Sera from individual monkeys were serially diluted and added to the coated plates. Captured ADA was detected with a horse radish peroxidase labeled anti-cynomolgus IgG antibody (Santa Cruz). The serum titer is defined as the reciprocal of the dilution factor required to give a 50% maximal signal in the ADA ELISA. ADA neutralizing activity was determined by incubating serum taken on day 46 after the first injection of C3201-HSA diluted to 1% in F12+1% BSA plus or minus 1 nM C3201 for 30 minutes at room temperature. The serum dilutions were added directly to serum-starved reporter cells prepared as described above in white poly-lysine coated 96 well plates. The plates were incubated for 4 hours after which the cells were lysed to measure luciferase activity with the Steady-Glo luciferase assay system (Promega). Luciferase activity was measured in relative luminescence units (RLU).

## Supporting Information

Figure S1 **Pharmacokinetic characterization of C3201-HSA in cynomolgus monkeys.** (A) Serum concentration of C3201-HSA in lean female cynomolgus monkeys following intravenous injection of 1 mg/kg C3201-HSA. C3201-HSA levels were determined using specific sandwich ELISA. Pharmacokinetic parameters for both monkeys, as well as the mean parameters are shown. (B) Serum concentration of C3201-HSA in lean female cynomolgus monkeys following subcutaneous injection of 2 mg/kg C3201-HSA. C3201-HSA levels were determined using specific sandwich ELISA. Pharmacokinetic parameters for both monkeys, as well as the mean parameters are shown.
(EPS)

Figure S2 **Analysis of anti-drug antibody in C3201-HSA in vivo functional study.** (A) Anti-drug antibody characterization in serum samples taken 2, 12, 26 and 69 days after first injection of C3201-HSA. Data are shown for each individual monkey, with antibody titer represented as the reciprocal of the binding EC50 for each given sample in the ADA detection ELISA. (B) Characterization of ADA C3201-neutralizing activity in a reporter cell assay. Data are shown for each individual monkey. Luciferase activity is measured in relative luminescence units (RLU). Sera taken from treatment groups block C3201 agonist activity while sera taken from the vehicle group do not.
(EPS)
